# Detection of Interfacial Structures in Inclined Liquid-Liquid Flows Using Parallel-Wire Array Probe and Planar Laser-Induced Fluorescence Methods

**DOI:** 10.3390/s20113159

**Published:** 2020-06-02

**Authors:** Lusheng Zhai, Zihan Meng, Jie Yang, Hongxin Zhang, Ningde Jin

**Affiliations:** School of Electrical and Information Engineering, Tianjin University, Tianjin 300072, China; lszhai@tju.edu.cn (L.Z.); mengzihan@tju.edu.cn (Z.M.); jyang@tju.edu.cn (J.Y.); zhxlixue@tju.edu.cn (H.Z.)

**Keywords:** liquid-liquid two-phase flow, interfacial characteristics, inclined pipe, laser-induced fluorescence, conductance parallel-wire array probe

## Abstract

Flows of two immiscible liquids through inclined pipes are often encountered in industrial processes. The interfacial characteristics in inclined pipes are of significance for understanding the mechanism of flow pattern transition and modeling the flow parameters. This paper developed a novel experimental technique to access the interface characteristics of liquid-liquid flows, during which optical and electrical methods were successfully combined by matching the refractive index and conductivity of the flows. A planar laser-induced fluorescence (PLIF) system was set up with a continuous laser and high-speed camera. Organic and aqueous phases were chosen to match refractive indices. The liquid-liquid interface in the middle of the pipe could be clearly visualized by the PLIF system. Meanwhile, two conductance parallel-wire array probes (CPAPs) were designed to reconstruct the liquid-liquid interfaces at upward and downward pipe cross-sections. The performances of the CPAP were validated using the PLIF results and employed to investigate the liquid-liquid interfacial structures. The interfacial shape and its instability were uncovered using the reconstructed interfaces by the CPAPs.

## 1. Introduction

Flows of two immiscible liquids through inclined pipes are often encountered in industrial processes [1,2,3]. For example, in the oil field, most wellbores have degrees of inclination from the horizontal flow due to the uncertain well trajectory. The pipe inclinations can produce quite different liquid-liquid interfacial structures compared to horizontal flows. The interfacial characteristics in inclined pipes are of significance for understanding the mechanism of flow pattern transition and modeling the flow parameters [4].

Pipe inclinations can lead to remarkable changes in liquid-liquid flow structures compared to horizontal flows [5,6,7]. The existing literature has mainly focused on the detection of flow patterns and pressure drop of liquid-liquid flows in inclined pipes. Lum et al. [8] identified oil-water flow patterns in upward (+5°, +10°) and downward (−5°) stainless steel pipes using a high-speed camera and electrical probes. They found that the minimum pressure drop occurred during the transition from dispersed water-in-oil to dual continuous flow. Rodriguez and Oliemans [9] conducted an oil-water flow experiment in a steel pipe (ID 8.28 cm) with different inclinations (−5° to +5°). The flow pattern boundaries were achieved via observation of recorded movies, during which a stratified wavy flow pattern with no mixing at the interface was identified in downward and upward flows. Hanafizadeh et al. [10] identified seven kinds of flow patterns in a 20-mm pipe using a high-speed camera and studied the effect of pipe inclination (−45 ° to +45°) on the transition boundaries between flow patterns. Kumara et al. [11,12] employed a particle image velocimetry to characterize the flow structures of oil-water flows in horizontal and slightly inclined stainless-steel pipes (ID 56 mm). Unfortunately, to date, no literature has reported the interfacial structures of the inclined liquid-liquid flows. Hence, it is not possible to comprehensively reveal the mechanism of the flow pattern transition, especially for the transition between stratified to non-stratified flows. 

Selected scholars developed physical models to predict the flow parameters of liquid-liquid flows in inclined pipes. Azizi et al. [13] presented an application of artificial neural networks in predicting the water holdup of oil-water two-phase flow in vertical and inclined pipes. Hanafizadeh et al. [14] predicted the pressure gradient in oil-water flows using homogeneous and two-fluid models and investigated the effect of the pipe inclinations on the prediction results. Grassi et al. [15] presented experimental pressure drops and flow-pattern map of oil and water with high viscosity ratio in horizontal and slightly inclined pipe to validate models in the literature, paying particular attention to the two-fluid model for annular flow and homogeneous no-slip model for oil-in-water dispersed flow. It should be noted that the close relations of the physical models are remarkably associated with the interface configurations of the flows [16,17,18]. The lack of knowledge about the interface characteristics can result in low prediction accuracy of the flow parameters. 

So far, several attempts have been made by scholars to detect the interfacial structures of stratified flows. Barral and Angeli [19] designed a double-wire conductance probe (DWCP) to study the fluctuating nature of the stratified interface in horizontal oil-water flows. The DWCP is sensitive to the interfacial structure at the center of the pipe but fails to indicate the interface shape at a whole pipe cross-section. As an extension of the DWCP, a multiwire capacitance probe was proposed by He et al. [20] to measure the stratified interface of gas-liquid flows. The multiwire capacitance probe enables the measurement of the liquid layer heights at different circumferential positions of the pipe. The wire-mesh sensor (WMS) is a newly developed technology of flow visualization and has been used in the measurement of the stratified interface of gas-liquid flows [21,22]. Note that the WMS performance in reconstructing the interface configurations remarkably depends on the temporal-spatial resolution of the sensor [23]. Besides, the ultrasonic technique has received increasing attention in two-phase flows due to its advantages of first response and no disturbance [24]. For example, Liang et al. [25] identified gas-liquid stratified flow, annular flow, and slug flow based on the ultrasonic pulse-echo signal from the inner pipe wall. However, the reconstruction of the stratified interface is difficult to implement due to the directivity limitations of the ultrasonic wave. 

Despite the previous research efforts, the investigation of the interface characteristics of inclined liquid-liquid flows still presents significant challenges. In this study, we implemented an experiment of liquid-liquid flows in inclined pipes (±3°). Optical and electrical methods were successfully combined in the experiment by matching the refractive index and the conductivity of the flows. A planar laser-induced fluorescence (PLIF) system was set up to visualize the liquid-liquid interface at the middle of the pipe. Meanwhile, two conductance parallel-wire array probes (CPAPs) were designed to measure the liquid-liquid interfaces at upward and downward pipe cross-sections. The CPAPs responses were validated by the PLIF visualizations and employed to reconstruct the liquid-liquid interface. The interfacial structure and its instability were uncovered using the CPAP data.

## 2. Experimental Setup

### 2.1. Inclined Flow Loop

**Figure 1:**
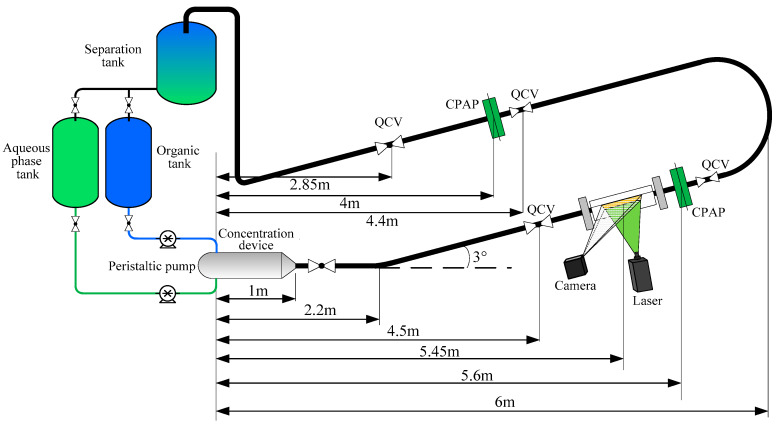
The experiment setup for the inclined liquid-liquid two-phase flows.

The experiment of inclined liquid-liquid two-phase flow was implemented in the multiphase flow laboratory of Tianjin University. The flow loop facility is shown in Figure 1. The test liquids were silicone oil and 54 wt% water/glycerol mixture. The glycerol was added into the water to match the refractive index of silicone oil. The fluid properties are shown in Table 1.

The two liquid phases were stored in their respective tanks and introduced into a concentration device, with the organic phase coming from the top and the aqueous phase from the bottom. The inner diameter and the length of the concentration device were 125 mm and 1000 mm, respectively. The concentration device can force the both liquids into a horizontal acrylic pipe with an inner diameter of 20 mm and a length of 1.2 m. Next, the liquids flowed into a +3° inclined pipe with a length of 3.8 m. After the upward pipe, the liquids entered a −3° downward pipe through a U-type connector. Finally, the liquids entered a gravity settler tank and were left to separate before they returned to their respective storage tanks. A PLIF system was set up on the upward inclined pipe. Two CPAPs and quickly closing valve (QCV) systems were respectively mounted on the upward and downward pipes.

### 2.2. Planar Laser-Induced Fluorescence (PLIF) System

**Figure 2:**
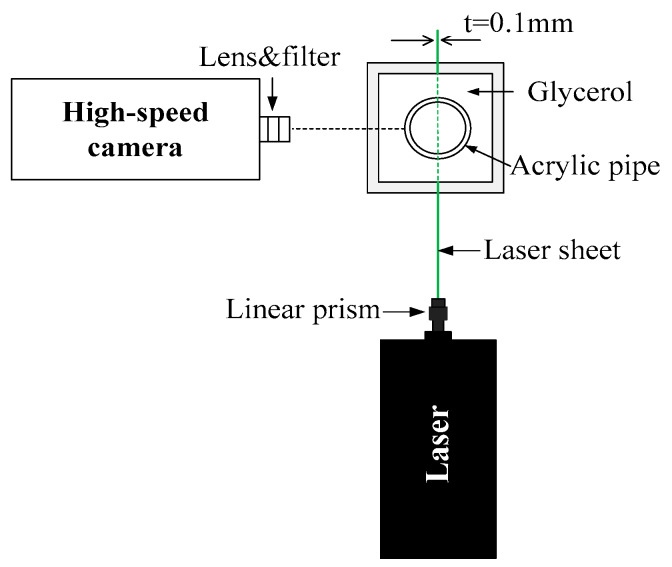
Sketch of planar laser-induced fluorescence system for flow visualization.

The setup for the PLIF experiment is shown in Figure 2. A view box filled with glycerol was located 5.45-m downstream the inlet. The filled glycerol allowed us to match the refractive index of the acrylic pipe, and thus minimized optical distortions at the curved pipe. A DPSS green continuous laser system (532 nm) by Laserglow Technologies was placed vertically below the pipe. The power of the laser was set as 3000 mW. A linear prism was used to form a laser sheet in the transverse direction and at the middle of the pipe. The thickness of the laser sheet was 0.1 mm at the focus position. Note that a fixed support was designed to adjust the laser with a +3° inclined angle and a distance of 50 mm to the view box according to its focal length.

The aqueous phase, i.e., water/glycerol mixture, was added to a small amount (0.02 ppm) of aqueous Rhodamine 6G, which can emit a light with wavelength higher than 590 nm under the excitation of the laser. A high-speed camera (FASTCAM Mini UX50, Photron, Japan) was placed on the side of the pipe, perpendicular to the visualization box, to record the flow images. A lens as a high pass filter (>580 nm) and 532-nm laser was used to remove the natural light. The frame frequency of the high-speed camera was selected as 1000 fps at a resolution of 1280*1024, and the shutter speed was set at 1/8000 s. According to the measurement principle of the PLIF, the brightness of the captured flow images can distinguish between the two liquid phases at a 2D plane and thus track the detailed liquid-liquid interfaces.

### 2.3. Conductance Parallel-Wire Array Probe (CPAP) System

Two conductance parallel-wire array probes (CPAPs) were designed to visualize the stratified liquid-liquid interfaces in inclined pipes. It should be noted that the conductivity of the aqueous phases was very low due to the addition of the glycerol in the tap water, leading to the invalidation of the conductance probes. A small amount of salt was added in the aqueous phase to increase the liquid conductivity to the level of tap water.

**Figure 3:**
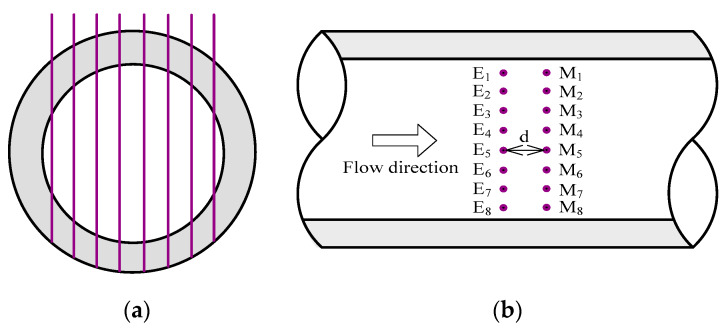
Sketch of the conductance parallel-wire array probe (CPAP) structures: (**a**) Front view for parallel-wire electrodes; (**b**) Top view.

The structure of the CPAP used in our experiment is shown in Figure 3. The CPAP consisted of eight pairs of parallel wire-type electrodes, i.e., E*_i_* and M*_i_*, *i* = 1, 2,…,8. E_1_ to E_8_ denote exciting electrodes and were equidistantly fixed at a pipe cross-section. M_1_ to M_8_ denote measuring electrodes and were equidistantly fixed at another pipe cross-section. The distance of the both pipe cross-sections was 2 mm, the diameter of the electrode was 0.2 mm, and the separation of electrodes at a same pipe cross-section was 2.5 mm.

**Figure 4:**
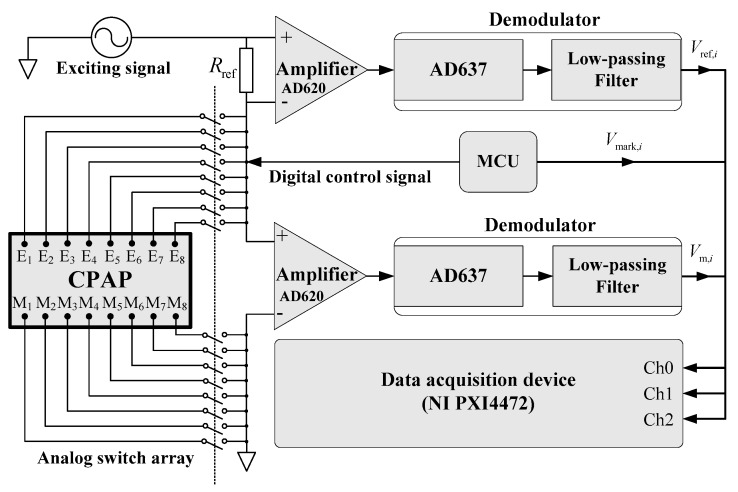
The sketch of the CPAP measure system.

The sketch of the CPAP measure system is shown in Figure 4. The CPAP works under a mode of cycle excitation [7]. During a certain time (0.625 ms), only electrode E_1_ was connected to an exciting signal *V*_e_ through a reference resistance *R*_ref_ (3 KΩ). The exciting signal was a sinusoidal voltage with a peak-to-peak value of 4 V, which was generated by an oscillator CG-402R2 under the control of a micro controller unit (MCU). The frequency of the exciting signal was selected as 20 KHz. Meanwhile, the measuring electrode M_1_ was connected to ground, and the MCU output a high-level signal, i.e., mark signal *V*_mark_. The voltage drops between the reference resistance *R*_ref_ and electrode pair E_1_-M_1_ were demonstrated by AD637, and accordingly, a reference signal *V*_ref,1_ and measuring signal *V*_m,1_ were produced by the demodulators. The signals of *V*_mark_, *V*_ref_,_1_ and *V*_m,1_ were simultaneously collected by a data acquisition card (PXI 4472, NI company, USA) to calculate the stratified interface height, *h*_1_, around the electrode pair E_1_-M_1_. Then, other electrode pairs were connected to the measure system successively by an analog switch array. After that, interface heights at eight different locations of a pipe cross-section were accessed, i.e., one frame data. The sampling frequency of the data acquisition card was set as 50 kHz/s/c to achieve a frame frequency of 200 fps.

Effective measuring signals of each electrode pair at every frame data were extracted and then averaged. The average values can be denoted by *V*_m*,i*_, *i* = 1,2,…,8. Similarly, the reference signal can be processed to obtain its average value *V*_ref,*i*_, *i* = 1,2,…,8. When the pipe is filled with tap water, the averaged values of measuring and referencing signals can be denoted by *V*_m,w,*i*_ and *V*_ref,w,*i*_, *i* = 1,2,…,8. Hence, the dimensionless conductance $G_{i}^{*}$ for the *i*th electrode pair can be given as:

(1) $$G_{i}^{*}=\frac{V_{\mathrm{ref},i}/V_{m,i}}{V_{\mathrm{ref},w,i}/V_{m,w,i}},\;i=1,2,\cdots ,8$$

The details about the calculation of dimensionless conductance were described in our previous publication [26].

**Figure 5:**
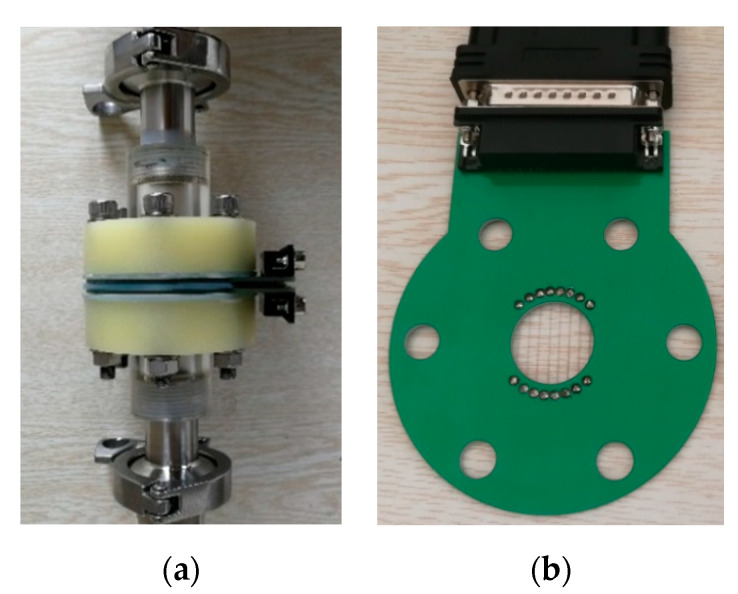
The conductance parallel-wire array probe used in the experiment: (**a**) CPAP; (**b**) Parallel electrodes.

The CPAP measurement system was calibrated using tap water and air. Figure 5 shows the CPAP used in the calibration experiment. Through gradually introducing tap water in a horizontal acrylic pipe using a syringe pump, flat water-gas interfaces with different heights were constructed, and at the same time, the responses of the CPAP were collected. According to Equation (1), the relationship between the dimensionless conductance,$G_{i}^{*}$, and the interface heights, *h_i_*, can be described by eight calibration curves:

(2) $$h_{i}=f_{i}(G_{i}^{*}),\;i=1,2,\cdots ,8,$$

According to the principle of the electrical method, a high water-air interface height can produce a high conductance of each electrode pair [19,27]. In the effective measuring zone of each electrode pair, the dimensionless conductance in the calibration experiment presents a liner increases tendency as the interface height is increased [7].

### 2.4. Visualization of Static Liquid-Liquid Interface

**Figure 6:**
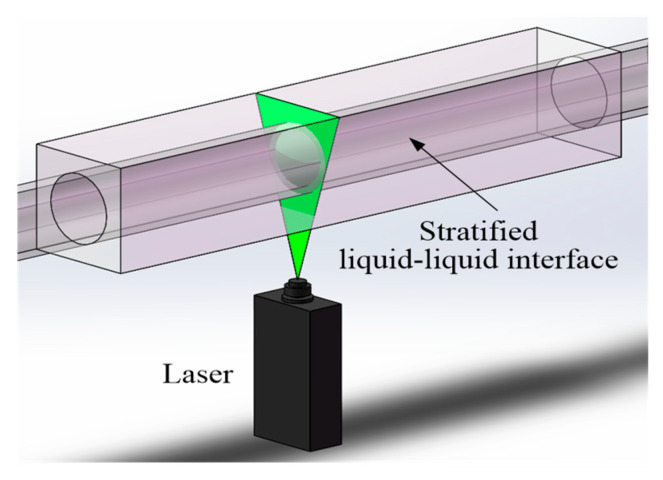
Sketch of the planar laser-induced fluorescence (PLIF) system for the visualization of the static liquid-liquid interface.

To validate the performance of the CPAP in detecting static liquid-liquid interface, as shown in Figure 6, a static experiment was carried out in a horizontal acrylic pipe. The properties of the experiment medium are shown in Table 1. Liquid-liquid interfaces with different heights were constructed and visualized using the PLIF system. With the purpose of visualizing the interface configuration, the laser sheet of the PLIF system was set parallel to the pipe cross-section. 

**Figure 7:**
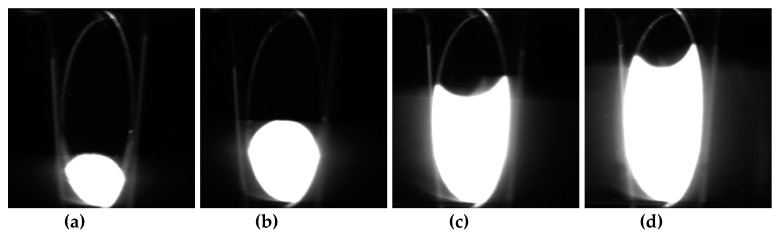
The static liquid-liquid interfaces visualized by the PLIF: (**a**) y_a_ = 20%; (**b**) y_a_ = 40%; (**c**) y_a_ = 60%; (**d**) y_a_ = 80%.

**Figure 8:**
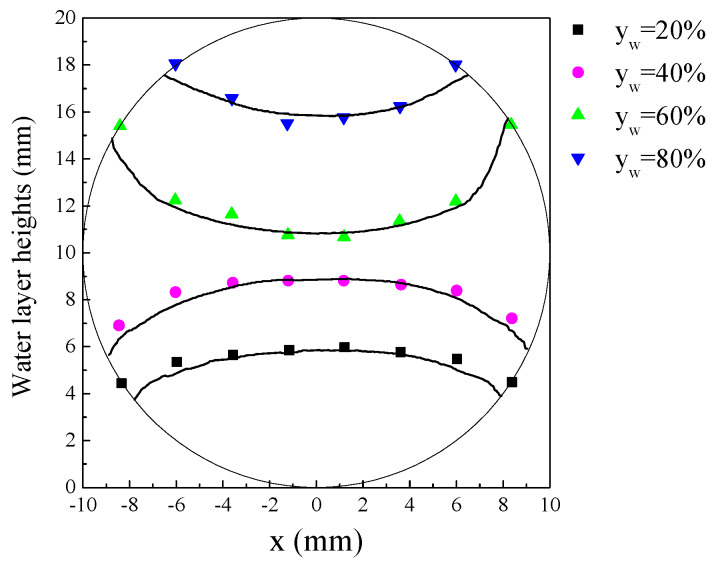
Comparison of static interface heights measured by the CPAP and PLIF. The solid lines represent the interface heights derived from the PLIF visualizations.

The PLIF visualizations of the liquid-liquid interfaces are shown in Figure 7. As can be seen, when the holdup, y_a_, of the aqueous phase was low, the liquid-liquid interface presented a convex shape. As the interface height increased, the interface shape changed to flat and then to concave. For each holdup of the aqueous phase, the CPAP responses were collected to calculate the dimensionless conductance using Equation (1). Then, the calculated dimensionless conductance was substituted into Equation (2) to access the interface heights at different locations of a pipe cross-section. In addition, the interface heights were derived from the PLIF visualizations by correcting the optical path distortion. Figure 8 shows the comparison of static interface heights measured by the CPAP and PLIF. As can be seen, the interfaces visualized by the PLIF agree well with the ones measured by the CPAP, indicating the good performance of the CPAP in measuring the liquid-liquid interface shapes.

## 3. Experimental Results

### 3.1. CPAP Responses

**Figure 9:**
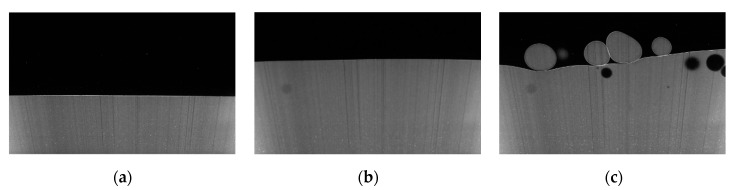
PLIF images for the flow conditions in the +3° inclined pipe: (**a**) Usa = 0.052 m/s, Uso = 0.15 m/s, ST flow; (**b**) Usa = 0.222 m/s, Uso = 0.15 m/s, ST flow; (**c**) Usa = 0.393 m/s, Uso = 0.15 m/s, ST&MI flow.

Figure 9 shows the PLIF images for different flow conditions in the +3° inclined pipe. As can be seen, a clear and stable stratified interface can be observed in Figure 9a,b, and this flow pattern was defined as stratified (ST) flow. Due to the increase of the flow rate, dispersed aqueous and organic droplets were observed around the stratified interface, as shown in Figure 9c, indicating the occurrence of the stratified flow with mixing at interface (ST&MI). Generally, the PLIF images present distinct interfaces between aqueous and organic phases, even for dispersed droplets, demonstrating the satisfactory performance of the PLIF system setup in this study.

**Figure 10:**
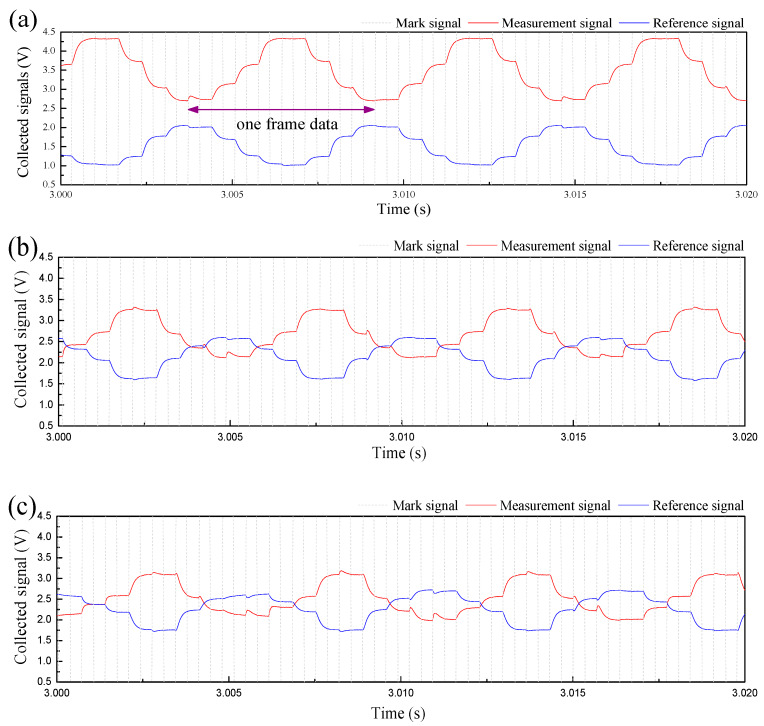
PLIF images for the flow conditions in the +3° inclined pipe: (**a**) Usa = 0.052 m/s, Uso = 0.15 m/s, ST flow; (**b**) Usa = 0.222 m/s, Uso = 0.15 m/s, ST flow; (**c**) Usa = 0.393 m/s, Uso = 0.15 m/s, ST&MI flow.

**Figure 11:**
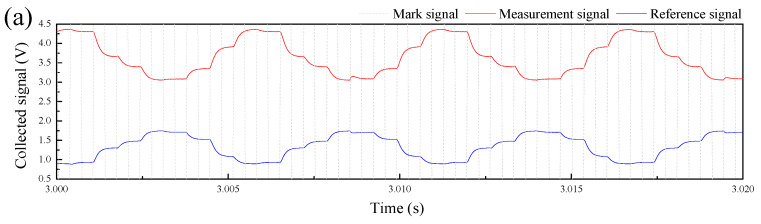

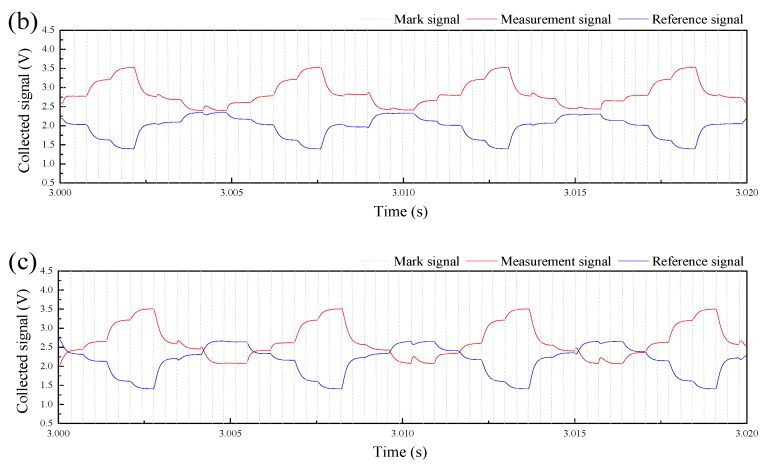
CPAP signals for the flow conditions in the −3° inclined pipe: (**a**) Usa = 0.052 m/s, Uso = 0.15 m/s, ST; (**b**) Usa = 0.222 m/s, Uso = 0.15 m/s, ST&MI; (**c**) Usa = 0.393 m/s, Uso = 0.15 m/s, ST&MI.

In the ±3° inclined pipe, the CPAP responses for typical flow conditions are respectively shown in Figure 10 and Figure 11. The mark signal, *V*_mark_, was a square wave changing from 0 to 5 volt, and it was employed to indicate the initiation of each frame data. The mark signals were only presented from 0.5 volts to 4.5 volts. Due to the short time shown in the X axis, the measurement and reference signals from the CPAP in a frame basically repeated the previous one. However, once the ST&MI flow occurred in the pipe, the signals collected from the eight electrode pairs indicated diversities due to the presence of the dispersed droplets. It should be noted that, for a constant flow rate of the organic phase, the measurement signal presented a decreasing tendency as the flow rate of the aqueous phase was increased, but it was opposite for the reference signal. The CPAP signals were utilized to derive the heights of the stratified interface and then reconstructed the interface structures using an interpolation operation. The reconstructed liquid-liquid interfaces are shown in Section 3.3.

### 3.2. Validation of CPAP Responses

The visualizations of liquid-liquid flow interfaces investigated in this study were focused on the ST and ST&MI flows. Considering the droplet entrainment in ST&MI flows, the investigation of the effect of the droplets on the CPAP responses is described in this section.

**Figure 12:**
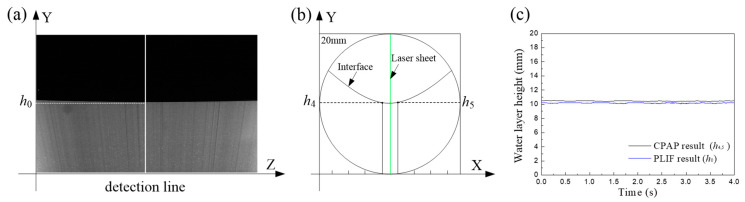
Interfacial heights at pipe center detected by the PLIF and CPAP: (**a**) Interfacial height extracted from the PLIF image; (**b**) Interface height detected by the CPAP; (**c**) Time-dependent series of the interface heights.

For a flow condition, as shown in Figure 12a, we used a multiscale edge detection method [28] to extract the interfacial height *h*_0_ at the middle of each PLIF image. The extracted interfacial heights were composed to a time-dependent series. As shown in Figure 12b, the interfacial heights measured by electrode pair 4 and 5 of the CPAP were respectively derived and denoted by *h*_4_ and *h*_5_. Their mean vale, *h*_4,5_, was calculated for comparison with *h*_0_, as shown in Figure 12c.

**Figure 13:**
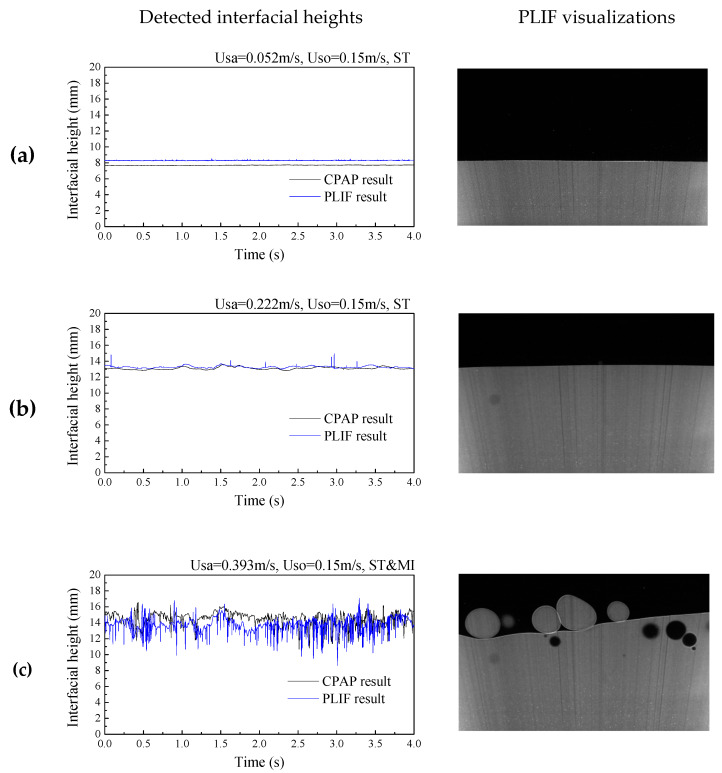
Comparison of the derived liquid-liquid interface heights by the PLIF and CPAP: (**a**) Usa = 0.052 m/s, Uso = 0.15 m/s, ST; (**b**) Usa = 0.222 m/s, Uso = 0.15 m/s, ST; (c) Usa = 0.393 m/s, Uso = 0.15 m/s, ST&MI.

Figure 13 shows the comparison of the derived liquid-liquid interface heights by the PLIF and CPAP. For ST flows, as shown in Figure 13a,b, the interfaces derived from the CPAP responses closely follow the PLIF results, even for the fluctuations of wavy interface. For ST&MI flows, as shown in Figure 13c, we can clearly observe the fluctuations of the interface and the droplets around it in the PLIF visualizations. Interestingly, the interfacial wavy characteristics and the entrained droplets detected by the CPAP indicate a good agreement with the PLIF result.

### 3.3. Interfacial Shape of Inclined Liquid-Liquid Flows

The analysis in the above section demonstrates the good agreement between the PLIF and CPAP results. The PLIF allowed for the visualization of the liquid-liquid interface at an axial 2D plane, but failed to indicate the interface structures at a pipe cross-section. In this section, reconstruction of the liquid-liquid interfaces in the positive and negative inclined pipes based on the CPAP responses is described.

**Figure 14:**
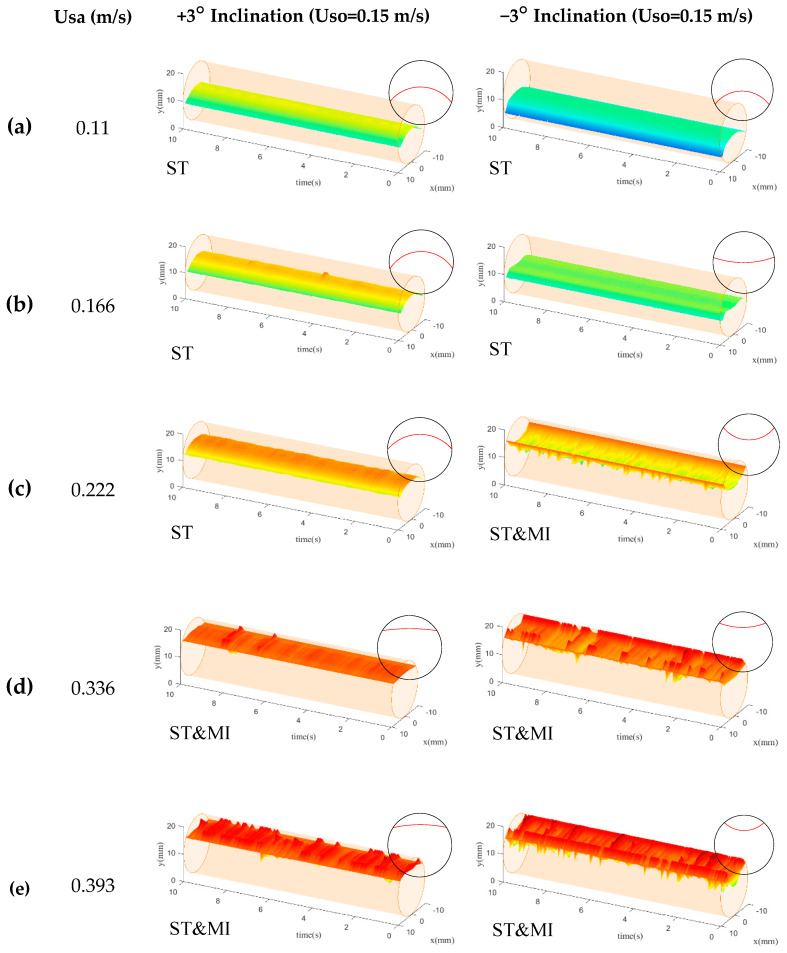
CPAP visualizations of the liquid-liquid interfaces for Uso = 0.15 m/s: (**a**) Usa = 0.11 m/s; (**b**) Usa = 0.166 m/s; (**c**) Usa = 0.222 m/s; (**d**) Usa = 0.336 m/s; (**e**) Usa = 0.393 m/s.

When the superficial velocity of the organic phase was equal to 0.15 m/s, as shown in Figure 14, the smooth liquid-liquid interface in ST flow was observed in the CPAP visualizations. Once the ST&MI flow occurred, the reconstructed interface became rough due to the presence of the droplets and interfacial fluctuations.

For both positive and negative inclinations, the interfacial height indicated an obvious increasing tendency as the flow rate of the aqueous phase was increased. Note that, for the flows in the −3° inclination, the heights of the liquid-liquid interface presented an obvious decrease, indicating that higher holdup values of aqueous phase were observed for upwardly inclined flows compared to the downwardly inclined flows. This result can be attributed to the slippage between the two phases.

The liquid-liquid interfaces in the +3° inclination were convex for the low flow rate of the aqueous phase, and then evolved to nearly flat shapes as the ST&MI flow occurred. However, for flows in the −3° inclined pipe, as the holdup of the aqueous phase increased, the liquid-liquid interface presented an evolution from convex to concave shape. Brauner et al. [29] and Ng et al. [30] proposed that the interface shape of horizontal liquid-liquid flows is determined by the Bond number, contact angle, and holdup. Notably, as shown in Figure 14, the interface fluctuations and droplet entrainment were not remarkable, and the contact angle on the pipe cross-section could be treated as constant. For a constant Bond number and contact angle, the liquid-liquid interfaces were always convex when the holdup of dense phase was low. As the holdup of the dense phase increased, the liquid-liquid interfaces turned flat and then concave [31], which is in line with the reconstructed interface by the CPAP. Thus, we can conclude that the interface shape for low total flow rate was mainly dominated by the holdup.

**Figure 15:**
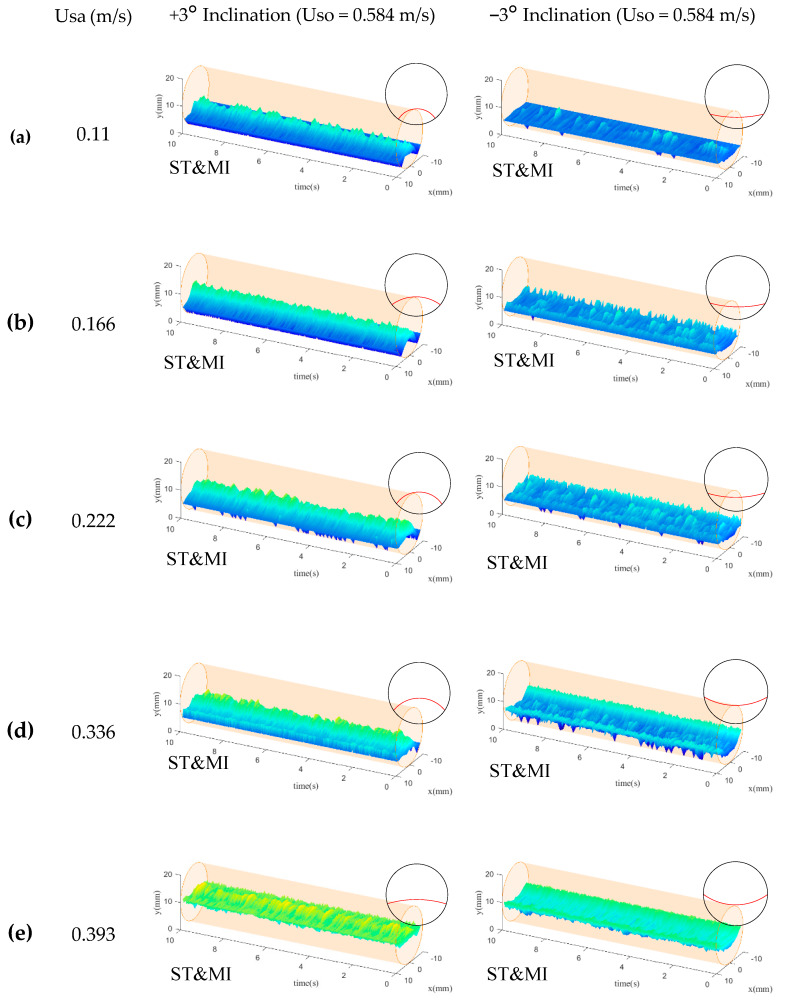
CPAP visualizations of the liquid-liquid interfaces for Uso = 0.584 m/s: (**a**) Usa = 0.11 m/s; (**b**) Usa = 0.166 m/s; (**c**) Usa = 0.222 m/s; (**d**) Usa = 0.336 m/s; (**e**) Usa = 0.393 m/s.

When the superficial velocity of the organic phase was increased to 0.584 m/s, as shown in Figure 15, the heights of the liquid-liquid interfaces in the positive and negative inclination pipes showed an obvious decrease due to the reduction of the aqueous phase holdup. Note that the interface heights in the −3° inclined flows were lower than those in +3° inclined flows, similar to the results shown in Figure 14.

It should be noted that, as shown in Figure 15, the liquid-liquid interfaces visualized by the CPAP presented irregular burr structures, which resulted from the unstable motions of the entrained droplets. The liquid-liquid interfaces in the positive inclination were mainly characterized by convex shapes, while the interfaces in the negative inclination present concave shapes. No obvious evolutions of the interface shape were encountered. Compared to the results shown in Figure 14, the interface shapes for high total flow rate were remarkably affected by the droplet entrainment rather than the holdup and contact angle.

The reconstructed liquid-liquid interfaces for the +3° and −3° inclined flows were characterized by positive and negative burrs, respectively, indicating the different directions of the droplet development. This result can be supported by the fact that, for +3° inclination, the organic phase flowed faster than the aqueous phase, and the aqueous phase was more easily stressed into droplets distributing in the organic phase. By comparison, for the −3° inclination, the velocity of the aqueous phase was higher than that of the organic phase, leading to the more droplets detaching from the organic phase.

### 3.4. Interfacial Instability of Inclined Liquid-Liquid Flows

**Figure 16:**
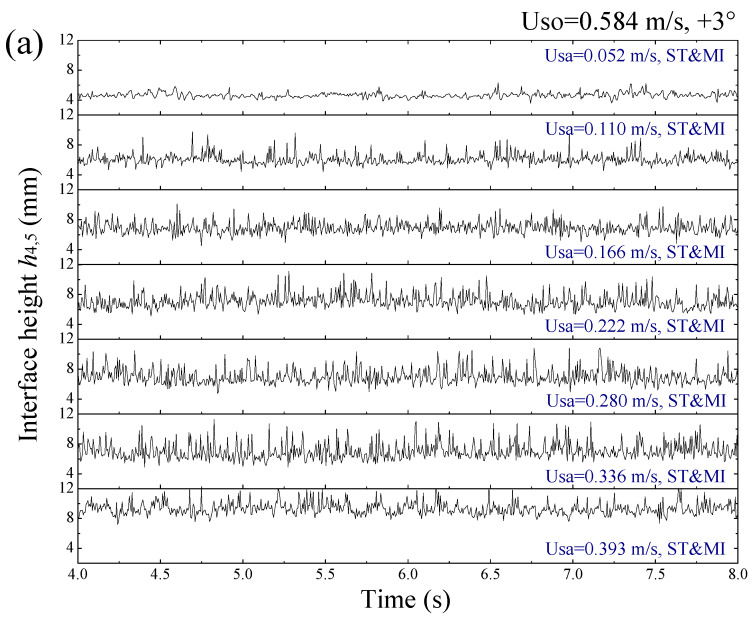

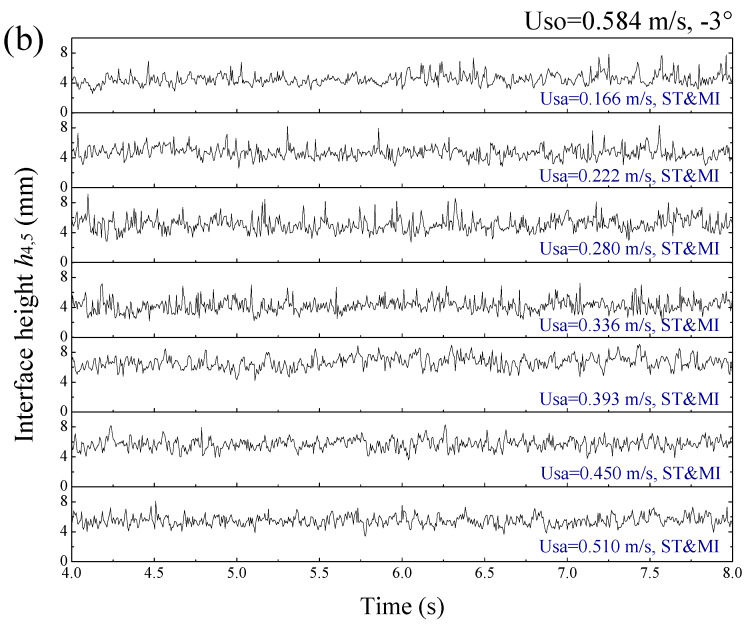
Fluctuating signals from electrode pairs 4 and 5: (**a**) Uso = 0.584 m/s, +3°; (**b**) Uso = 0.584 m/s, −3°.

The motions of the stratified liquid-liquid interface and mixing layer resulted in complex fluctuations of the probe signals, as shown in Figure 16. For a constant flow rate of the organic phase, the signal fluctuations became more and more remarkable as the flow rate of the aqueous phase was increased. Note that the signal fluctuations for the upwardly and downwardly inclined flows presented obvious differences. The interfacial characteristics of the flows were implied in the probe responses and externalized as short-term fluctuations of the signals. As an effective way to qualify short-term properties of time series, the Poincaré plot was employed in this study to explore the interfacial instability of the inclined liquid-liquid flows based on the probe signals.

**Figure 17:**
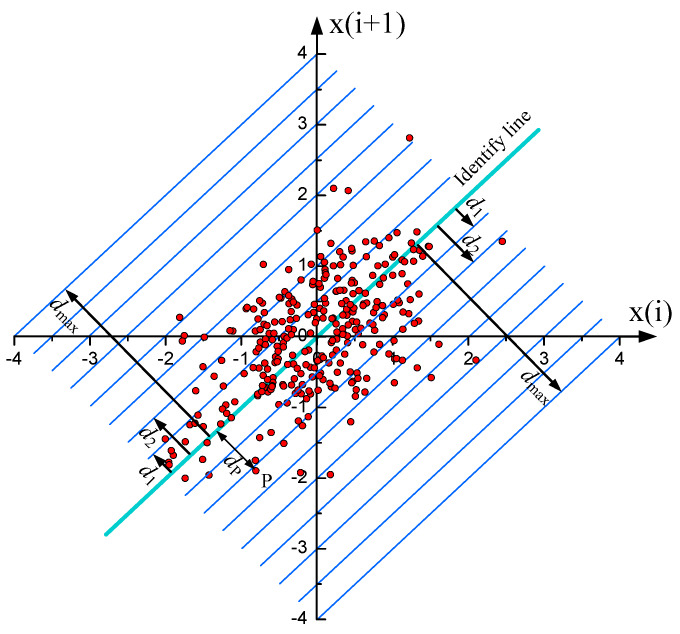
Poincaré plot for the two-dimensional graphical representation of time series.

The Poincaré plot is a two-dimensional graphical representation of temporal correlations within adjacent values of a time series {*x_i_*}, *i* = 1,2,…,*n* [32,33]. On the Poincaré plot, as shown in Figure 17, the current and next values, *x_i_* and *x*_*i*+1_, of time series are represented on x-axis and y-axis, respectively. Thus, each point (*x_i_*, *x*_*i*+1_) on the Poincaré plot corresponds to two successive values of the time series.

**Figure 18:**
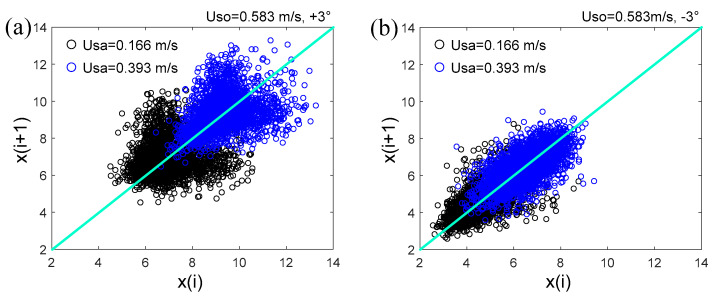
Poincaré plot of the probe signals collected from typical flow conditions: (**a**) Uso = 0.584 m/s, +3°; (**b**) Uso = 0.584 m/s, −3°.

Figure 18 shows the Poincaré plot of the probe signals collected from typical flow conditions. As can be seen, the scattered points on the Poincaré plot basically showed a symmetrical distribution about the identify line. The topological structures of the scattered points on the Poincaré plot were sensitive to the changes of the flow conditions. The distributions of the scattered points were associated with the instability of the wavy interface and mixing layer of the droplets. Thus, a measure, i.e., short-term distribution entropy, was used to characterize the instable interface.

As shown in Figure 17, we divided the Poincaré plot into subregions using 2*N* lines, which were parallel with the identity line. The distance between the kth line and the identity line was denoted as:

*d_k_* = *kd*   (1 ≤ *k* ≤ *N*) (3)

where *d = d_max_/N* is the separation of the adjacent parallel lines, and *d_max_* is the maximum distance of the points to the identify line. Hence, the signal fluctuations corresponding to the interfacial dynamics were quantified into *N* levels of short-term variability. We countered the number of the points in each subregion, and then calculated its ratio to all the points on the Poincaré plot, i.e., *p_i_*, *i* = 1,2, …, N. Thus, we can define short-term distribution entropy (SDE) on the Poincaré plot as

(4) $$\mathrm{SDE}=-\sum_{i=1}^{N}p_{i}\log_{2}p_{i}$$

A high SDE can indicate high instability of the liquid-liquid interface.

**Figure 19:**
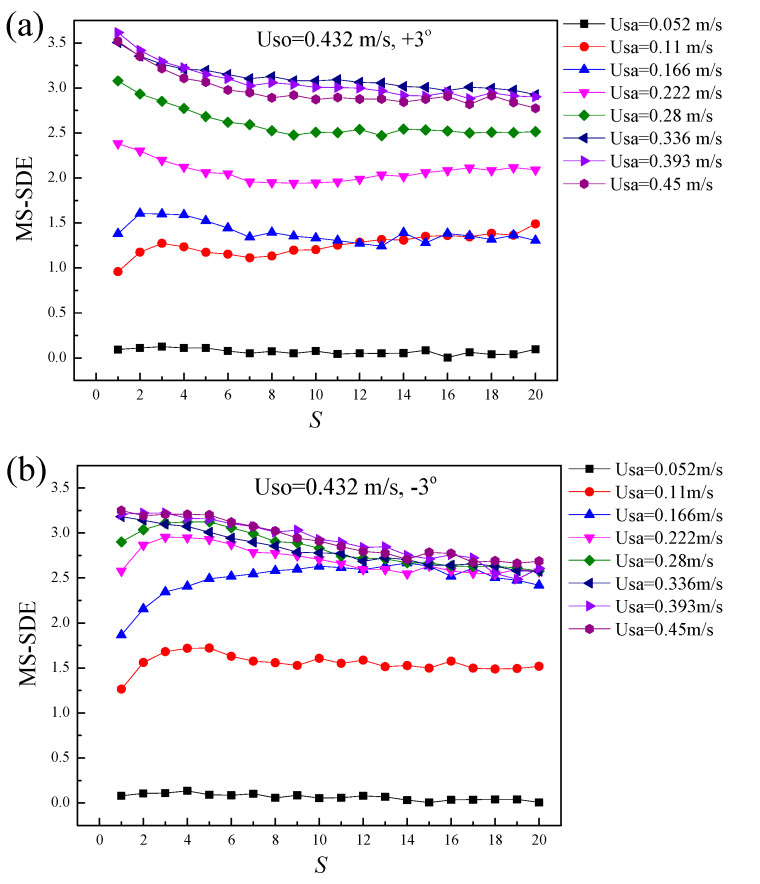
The multiscale short-term distribution entropy (MS-SDE) for the inclined liquid-liquid flows: (**a**) Uso = 0.432 m/s, +3°; (**b**) Uso = 0.432 m/s, −3°.

Complex dynamics of nonlinear systems always imply multiple time scales [34,35]. For a given one-dimensional time series {*u_t_*}, *t* = 1,2,…, *m*, a consecutive coarse-grained time series $\left\{x_{i}^{\tau }\right\}$ can be constructed as

(5) $$x_{i}^{\tau }=\frac{1}{\tau }\sum_{t=(i-1)\tau +1}^{i\tau }u_{t}\;1\leq i\leq n,\;n=\left[\frac{m}{\tau }\right]$$

where *τ* denotes the time scale, and [*m*/*τ*] denotes the largest integer less than or equal to *m*/*τ*. For each coarse-grained time series $\left\{x_{i}^{\tau }\right\}$, a Poincaré plot can be obtained to access the SDE. Through changing the values of the time scale *τ*, we calculated the SDE at different time scales, i.e., multiscale short-term distribution entropy (MS-SDE). Figure 19 shows the MS-SDE of inclined liquid-liquid flows with the organic phase velocity of 0.432 m/s. As can be seen, the MS-SDE depends on the time scales and the changes of the flow conditions. Generally, for a constant velocity of the organic phase, a higher velocity of the aqueous phase can produce a larger SDE.

**Figure 20:**
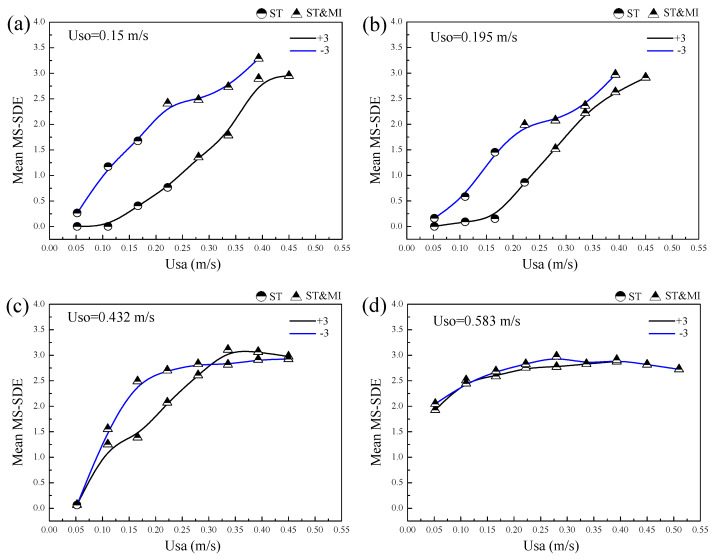
Mean MS-SDE of probe signals for the inclined liquid-liquid flows: (**a**) Uso = 0.15 m/s; (**b**) Uso = 0.195 m/s; (**c**) Uso = 0.432 m/s; (**d**) Uso = 0.583 m/s.

The MS-SDE calculated for the ST and ST&MI flows in the inclined pipes were averaged over the time scales, as shown in Figure 20. The mean MS-SDE for ST flow was very low, since the interface of the ST flow was smooth and the interfacial fluctuation was not remarkable. When the ST&MI flow occurred, however, the MS-SDE indicated an obvious increase. For a constant flow rate of the organic phase, a high velocity of the aqueous phase produced a high MS-SDE, indicating the intensified instability of the interfacial fluctuation and the droplet mixing layer.

Note that, for low velocities of the organic phase, as shown in Figure 20a–c, the liquid-liquid interface in the downwardly inclined flows was more unstable compared to the upwardly inclined flows. However, when the velocity of the organic phase was increased to 0.583 m/s, as shown in Figure 20d, the interfaces of the upwardly and downwardly inclined flows showed similar instability. As mentioned in Section 3.3, for a high velocity of the organic phase, the flows in the positive inclination were mainly characterized by the droplets detaching from the aqueous phase, but for negatively inclined flows, the opposite was true. The result shown in Figure 20d demonstrate that even though the directions of the droplet development in liquid-liquid flows were different for positive and negative inclinations, they exhibited similar contribution to the interfacial instability.

## 4. Conclusions

This paper developed a novel experiment technique to access the interface structures of inclined liquid-liquid two-phase flows, during which optical and electrical methods were successfully combined. A planar laser-induced fluorescence (PLIF) system was set up with a continuous laser and high-speed camera. The liquid-liquid interfaces at the middle of the pipe could be clearly visualized by the PLIF system. Meanwhile, two conductance parallel-wire array probes (CPAPs) were designed to reconstruct the liquid-liquid interfaces at upward and downward pipe cross-sections. The performances of the CPAPs were validated by the PLIF visualizations. It was found that the CPAPs allowed for the detection of the liquid-liquid stratified interface and were sensitive to the appearance of the droplets.

For a low flow rate of the organic phase, the liquid-liquid interfaces were regular and presented an evolution process from convex to concave shape with the flow rate of aqueous phase increasing. The interface shape was mainly dominated by the liquid holdup. In comparison, if the flow rate of the organic phase was high enough, the liquid-liquid interfaces presented irregular structures because of the unstable motions of the entrained droplets. The liquid-liquid interfaces in positive inclination were mainly characterized by convex shapes, while the interfaces in negative inclination presented concave shapes. No obvious shape evolution behavior of the interface was investigated for the high flow rate of the organic phase, and the liquid-liquid interface shape was dominated by the droplet entrainment.

Two-dimensional graphical representations, i.e., Poincaré plots, were constructed using the CPAP signals. Multiscale short-term distribution entropy (MS-SDE) was derived from the Poincaré plot to indicate the interfacial instability. The interfacial instability was affected by the pipe inclination as well as the flow rate. The liquid-liquid interface for the −3° inclined flows always indicated high instability. However, if the flow rate of the organic phase was high enough, the interfaces of the upwardly and downwardly inclined flows showed close instability.

## Figures and Tables

**Table 1:** Fluid properties of the liquid-liquid experimental system at 20 °C.

Phase	Liquid	$\rho {\;(\mathrm{kg}/m}^{3})$	μ (mPas)	Refractive Index	Conductivity (μS/cm)
Aqueous *	Water /Glycerol	1033	7.9	1.4	396.8
Organic	Silicone oil	963	4.8	1.4	-

* A small amount of Rhodamine 6G and salt were added in aqueous phase. The water in aqueous phase was tap water.
